# Oral Pemphigus Vulgaris Treatment with Corticosteroids and Azathioprine: A Long-Term Study in Shiraz, Iran

**DOI:** 10.1155/2022/7583691

**Published:** 2022-09-17

**Authors:** Mehdy Davarmanesh, Maryam Zahed, Asma Sookhakian, Sina Jehbez

**Affiliations:** ^1^Department of Oral and Maxillofacial Medicine, School of Dentistry, Shiraz University of Medical Sciences, Shiraz, Iran; ^2^Oral and Dental Disease Research Center, Department of Oral and Maxillofacial Medicine, School of Dentistry, Shiraz University of Medical Sciences, Shiraz, Iran; ^3^Student Research Committee, School of Dentistry, Shiraz University of Medical Sciences, Shiraz, Iran

## Abstract

**Background:**

Treating oral mucosal lesions of Pemphigus Vulgaris (PV) disease is usually challenging for clinicians. We studied the treatment outcomes of the oral PV patients referred to the Oral Medicine Department of Shiraz University of Medical Sciences from 2004 to 2018.

**Methods:**

The medical records of 54 oral PV patients with histopathological confirmation who were treated by a single protocol were studied. The protocol consisted of initial treatment with 1 mg/kg/day of oral prednisolone for all patients. After 4–6 weeks, all patients were prescribed 40 mg of prednisolone. If lesion recovery was not observed or new lesions had developed, adjuvant therapy (maximum dose of 200 mg per day of Azathioprine (AZA)) was initiated anytime during the treatment. The oral prednisolone dosage was gradually tapered to 5 mg/alternate day in 9 months.

**Results:**

47 patients were included in the study. 34.04% were male and 65.96% were female with a mean age of 41.83 ± 12.520. The mean follow-up period was 50.806 ± 44.417 months (over 4 years). The severity of oral involvement was mild in 21.27%, moderate in 36.17%, and severe in 42.6%. During treatment, all patients except one experienced complete remission. The mean time to achieve complete remission was 150.39 ± 224.075 days. Most of the patients experienced relapse due to self-discontinuation of treatment. 55% had complete remission and 43% were in partial remission at the last follow-up session. In 65.96% of patients, treatment-associated side effects were observed. The patients treated with prednisolone alone had significantly more side effects than those using AZA as an adjuvant (80% vs 50%, respectively; *P*=0.030). The mean duration of follow-ups was longer for patients with side effects (*P* < 0.01). Topical corticosteroids were used for all patients sometime during the treatment. No deaths were recorded.

**Conclusion:**

Prescribing low-dose prednisolone and adding AZA in nonresponding cases has good clinical outcomes for the treatment of oral lesions of PV. Adjuvant therapy can avoid the increase in corticosteroid dosage and side effects. The treatment method described in this study can be a helpful guide for clinicians, especially when other immunosuppressive drugs are not available.

## 1. Introduction

Pemphigus is a group of chronic blistering autoimmune diseases involving cutaneous and mucosal membranes [1]. Pemphigus Vulgaris (PV) is the most prevalent subtype of pemphigus, comprising up to 70% of all cases [2]. The estimated worldwide annual incidence of PV is 1–5 per 1000000 populations [3, 4]. The occurrence is most common in middle-aged and older adults between the fifth and sixth decades of life [4]. In addition, it has a male-to-female ratio of 1 : 2, showing a higher incidence in women [4]. Although both genetic and environmental factors such as pesticides and oral contraceptives play a role in the etiopathogenesis of PV, a strong genetic background is mentioned due to more frequent occurrences in certain racial groups such as Ashkenazi Jews and higher occurrence in regions like Brazil [3–5].

The first site of involvement in two-thirds of PV patients is the oral mucosa [6, 7]. Nevertheless, in most cases in which cutaneous lesions develop first, oral lesions will ultimately arise [6, 8]. The mortality rate of PV has decreased in recent years due to therapeutic advances [9], but it is still an important cause of significant mortality [5]. Therefore, PV treatment has been a challenge during the past decades and continues to be so [10]. Moreover, the treatment of oral PV is mostly recalcitrant and challenging due to the chronic nature of the disease and the rough oral environment [6]. Recurrences are common due to the lack of patient cooperation and poor oral hygiene. Also, the use of dental prostheses and dental restorations, oral habits, smoking, and alcohol consumption can complicate the management [6, 11, 12].

Previous studies showed that immune imbalance and antibody generation were the key factors to the start of active pemphigus. Patients with acute pemphigus had elevated levels of circulating IL-17+, TH17, TFH17, and TFH17.1 cells. Elevation in levels of TH17 and TFH17 cells correlated with higher levels of Dsg-specific CD19+CD27+ memory B cells, and, therefore, patients with active pemphigus demonstrated higher levels of Dsg3-autoreactive TFH17 cells [13].

PV treatment is based on immunosuppression. Corticosteroids have been the mainstay of treatment of PV since the time of their approval in the 1950s [14]. Although topical corticosteroid therapy is used in cases where the PV is not extensive, and lesions are exclusively oral, systemic corticosteroids are the mainstay of treatment of oral lesions [15, 16]. The optimum corticosteroid dosing and tapering strategies are unknown. However, we know that prolonged, high-dose administration of systemic corticosteroids has severe adverse effects, including hypertension, osteoporosis, atherosclerosis, peptic ulcer disease, aseptic necrosis, diabetes mellitus/glucose intolerance, susceptibility to infections, and septicemia [17, 18]. On the other hand, if corticosteroids cannot control the condition solely or when the patient has clinical contraindications to high-dose corticosteroids, other drugs, called adjuvant or corticosteroid-sparing agents, should be administered simultaneously [10]. Azathioprine (AZA) is the oldest and most prescribed immunosuppressive medication for autoimmune bullous disease, particularly in PV [19, 20]. Its efficacy as an adjuvant is well documented and was first used successfully in 1969 [17, 20].

Recent reports mentioned that it is difficult to compare different adjuvant drugs in terms of treatment outcomes for PV patients due to the lack of well-designed long-term studies [15, 20]. Furthermore, despite the high frequency of oral involvement in PV patients, there are few long-term studies regarding the management of oral lesions. Delay in diagnosis by dentists is also a major complication in the management of such patients [15]. A long-term cohort of oral PV patients showed that the treatment strategy remains patient-specific and need-based [15]. Therefore, further studies which show the long-term management of oral PV are yet needed.

This study aimed to evaluate the long-term treatment outcomes and side effects of a treatment protocol combining low-dose corticosteroids and adjuvant therapy with AZA. This report can be a helpful guide for future researchers or clinicians to manage oral PV patients, especially patients who are nonresponsive to systemic corticosteroids alone or in regions where rituximab is not freely available.

## 2. Materials and Methods

### 2.1. Patients

This cross-sectional retrospective study was conducted on the medical records of PV patients with oral manifestations who visited the Oral Medicine Department of Shiraz University of Medical Sciences from 2004 to 2018.

The following data were extracted from the files: gender; age; medical history; medications; time of disease onset; disease severity; distribution of oral lesions; involvement of extraoral sites; treatment strategies (dosage, tapering, intralesional injections); number of follow-ups; treatment-associated side effects; time of relapse; number of relapses; time to achieve complete remission; partial remission; and corticosteroid dosage at disease remission, relapse, and end of treatment. To note, all patients had signed an informed consent form before the start of treatment and this study was approved by the ethics committee of the Shiraz University of Medical Sciences (IR.SUMS.DENTAL.REC.1398.077).

### 2.2. Inclusion Criteria

1. Patients with oral PV who were confirmed by clinical, histological, and direct immunofluorescence staining in the same laboratory
2. Patients who were treated by the same protocol and managed by a single clinician who had observed the patients in all follow-up sessions

### 2.3. Exclusion Criteria

1. Medical records with incomplete data
2. Patients who were treated by different clinicians and under different protocols
3. Patients who had received other treatments for extraoral sites
4. Patients with no follow-up sessions

From the total of 142 oral PV records, 54 were treated by the same clinician. After excluding incomplete records, a total of 47 medical records were included in this study. In other words, 7 medical records were excluded because of referral to dermatologists (*N* = 1), incomplete follow-ups (*N* = 5), and topical corticosteroid therapy with no systemic treatments (*N* = 1).

### 2.4. Treatment Method

**Figure 1:**
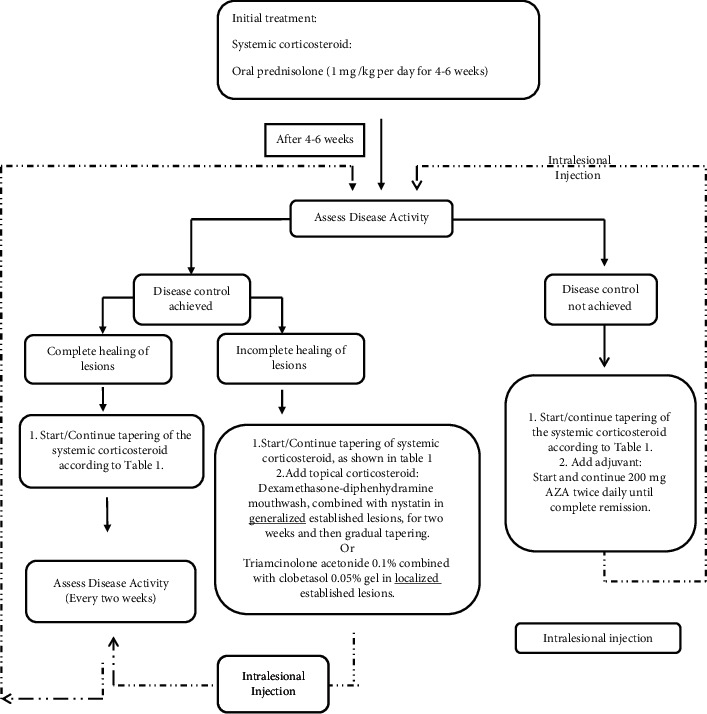
Flowchart of the treatment protocol.

Patients were initially treated with oral prednisolone as a systemic corticosteroid with starting dose of 1 mg/kg/day. Thereafter, disease activity was assessed after 4–6 weeks of corticosteroid therapy. In order to avoid high-dose corticosteroid side effects, all patients were put on a 40 mg prednisolone regimen, regardless of the initiating dose, in this follow-up session. Nonetheless, if lesion recovery was not observed or new lesions had developed, adjuvant therapy (maximum dose of 200 mg per day of AZA) was initiated, in addition to the oral prednisolone any time during the treatment. The oral prednisolone dose was gradually tapered down in 9 months for patients under clinical control every 4 weeks to minimize side effects based on the tapering schedule seen in Table 1. To note, intralesional corticosteroid was injected in the follow-up sessions for cases of PV with persistent oral lesions. All patients received topical corticosteroids in the form of an ointment, paste, or mouthwash next to topical antifungal therapy (nystatin oral drop) sometime during the treatment. An overview of the treatment protocol is shown in Figure 1.

During the treatment, a clinical oral examination was performed and blood pressure was recorded every 2 weeks. All patients were monitored by laboratory parameters such as complete blood count, liver function test, renal test, and fasting blood sugar every month. They were also recommended to switch to a diet low in calories, salt, and carbohydrate and to increase the intake of potassium and vegetables to control weight gain, hypertension, and hyperglycemia. Due to corticosteroid-induced osteoporosis, calcium (500 to 1000 mg per day) and vitamin D (200–400 IU per day) were administered as dietary supplements. Moreover, dual-energy X-ray absorptiometry (DEXA scan) was performed for all patients to measure bone density every 6 months. Osteopenic patients were referred to medical centers and internists in order to receive other medications such as intranasal calcitonin and oral bisphosphonates. During this period, patients were also referred to an ophthalmologist, dermatologist, and internist for the detection of extraoral lesions. If liver dysfunction or myelotoxicity was observed in the group receiving AZA, the dosage was reduced to 100 mg daily.

Regarding latent infections such as HIV, hepatitis B and C, tuberculosis, and other acute and chronic infectious diseases, the patients and their family members in close contact with the patients were monitored before and during the treatment. The patients were asked to monitor both themselves and their family members closely for changes suggestive of acute and chronic infections. Informative leaflets and pamphlets regarding infectious diseases were handed out to the patients for better self-monitoring.

### 2.5. The Terms Used

Baseline. It was defined as the first visit of each patient to the Department of Oral Medicine.

Disease Severity. It was rated based on the extent of oral involvement as follows: mild (for the involvement of ≤25%), moderate (for the involvement of >25% and <75%), and severe (for the involvement of ≥75%).

Control of Disease Activity. It was defined as the time at which new lesions ceased to form and established lesions began to heal following a consensus statement on definitions of disease activity, endpoint, and therapeutic response for PV [9].

Time to Complete Remission. It was calculated from the onset of treatment to the first follow-up session at which no new lesions had developed and all established lesions had healed.

Partial Remission. It was defined as the disease being under clinical control with topical corticosteroid in addition to the small dose of systemic corticosteroid.

Relapse/Flare. It was defined as the appearance of new lesions or extension of established lesions in a patient who had achieved disease control.

### 2.6. Statistical Analysis

The data derived from the records were analyzed by the statistical software of SPSS 2018 (Statistical Package for the Social Sciences). The Kruskal–Wallis test, Mann–Whitney *U* test, and chi-squared test were used for the comparison of data. A *P* value of <0.05 was considered significant.

## 3. Results

### 3.1. Patients' Characteristics

Of all 47 patients, 16 (34.04%) were male and 31 (65.96%) were female with the age range of 20–75 years (mean 41.83 ± 12.520). The duration of oral involvement at baseline was 30–1095 days (mean value ± SD = 216.89 ± 232.647). 19 patients had a history of primary systemic disease. The medical history of the patients included breast cancer, osteoporosis, hypertension (*N* = 2), palpitations, hyperlipidemia (*N* = 2), diabetes mellitus, rheumatoid arthritis, G6PD deficiency, Gilbert's syndrome, dysuria, nephrolithiasis, migraine, psychological problems, reflux, and respiratory problems. In addition, balloon angioplasty was performed in one of the study patients. The familial history of the patients included cancer (*N* = 3), diabetes (*N* = 2), hyperthyroidism (*N* = 1), autoimmune disease (*N* = 1), and PV (*N* = 1). In terms of pharmacological history, 16 patients were on medications such as captopril, propranolol, atorvastatin, rosuvastatin, glibenclamide, finasteride, diphenhydramine, diazepam, alprazolam, citalopram, nitroglycerin, aspirin, clopidogrel, ticlopidine, colchicine, pantoprazole, fluconazole, tetracycline, levofloxacin, and folic acid. There was no history of latent infectious disease in the patients and their family members before treatment and during the treatment.

**Figure 2:**
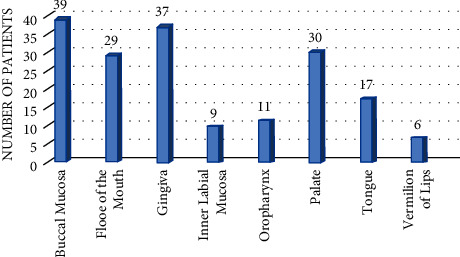
Distribution of oral lesion in PV patients.

Clinically, all 47 patients had oral lesions. The severity of oral involvement was mild in 10 (21.27%), moderate in 7 (36.17%), and severe in 20 (42.6%) patients. The buccal mucosa was the most common site of oral involvement as shown in Figure 2. On the other hand, extraoral involvement was also seen in 31 of 47 patients (65.96%). The most common site of extraoral involvement was the nasal mucosa.  Table 2 describes other characteristics of the study population.

### 3.2. Treatment Outcomes

25 patients were treated with oral prednisolone, and 22 were treated with oral prednisolone plus AZA. There was no significant difference among these two treatment groups regarding sex and age (*P* > 0.05). However, disease severity was greater significantly in the prednisolone plus AZA group in comparison to the patients who used prednisolone with no adjuvant (*P*=0.035) (Table 3).

According to the patient records, the mean body weight of the patients was 65.13 ± 10.234 kg. The mean body weight of patients treated with corticosteroids alone (group 1) was 62.67 ± 13.300 kg and for individuals receiving AZA (group 2) was 68.76 ± 13.300 kg. The mean initial dose of oral prednisolone was 60.21 ± 11.130 mg. All patients also used a topical corticosteroid as a concomitant medication during their treatment, except one patient in the prednisolone group. The number of follow-up sessions was 2–48 times during the evaluated years (mean value ± SD = 19.26 ± 11.789), and the duration of follow-ups was 45–5171 days (mean value ± SD (range) = 1524.18 ± 1332.514). A summary of demographic and clinical characteristics, medical history, follow-up data, and treatment outcomes is reported in Tables 2 and 3.

During treatment, all patients except one experienced complete remission. However, 36 patients suffered at least one relapse (23 had one relapse and 13 patients had two relapses). Relapse in 20 patients was mainly due to self-discontinuation of therapy. The most commonly involved site at the time of relapse was the gingiva. To note, prednisolone dosage and topical corticosteroids were adjusted in cases of relapse according to the severity of relapse and steroid dosage at the time. The overall evaluation of the long-term treatment outcomes of our study population is reported in Table 3.

According to the statistical analysis, the duration of oral involvement at baseline was not found to be correlated significantly with the disease severity (*P*=0.340), time to achieve a complete remission (*P*=0.128), and the oral prednisolone dose at the end of treatment (*P*=0.753). In addition, the disease severity did not correlate significantly with the oral prednisolone dose at the time of complete remission (*P*=0.901) and the time to achieve complete remission (*P*=0.800).

### 3.3. Treatment-Associated Side Effects

Treatment side effects were observed in 31 patients (65.96%). The most frequent side effects were mild hypertension, followed by gastrointestinal problems and insomnia. Other observed side effects included ageusia, blurred vision, epistaxis, hot flash, vitiligo, pruritus, liver dysfunction, nutritional problems, respiratory problems, osteoporosis, acne, irritability, anxiety, weakness, facial edema, and other cushingoid features. According to our records, the maximum number of side effects observed in one patient was six. No sign of myelotoxicity was observed. To note, all side effects were diminished after corticosteroid and AZA dosage reduction. There was a significant difference in the rate of patients with side effects in the prednisolone group vs prednisolone plus AZA group (80% vs 50%, respectively; *P*=0.030) (Table 4).

The mean duration of treatment and follow-ups in days was longer in the group of patients with side effects compared to the group of patients without side effects: 2141.48 (± SD 1277.974, range: 193–5171) vs 405.31 (± SD 296.128, range: 45–1185). A statistically significant difference between these values was recorded (*P* < 0.01).

## 4. Discussion

Prednisone and prednisolone are the most commonly used corticosteroid drugs in the treatment of PV [15, 21]. They have potent anti-inflammatory and immunosuppressive characteristics and affect almost every aspect of the immune system [15, 22]. The most important immunosuppressive outcome of corticosteroids is the effect they have on T-cell activation. They inhibit cytokine release and thus reduce molecule production. They also induce transient lymphocytopenia by altering lymphocyte recirculation and lymphocyte death [22]. AZA is a synthetic purine analogue that is metabolized to 6-mercaptopurine and its active metabolite 6-thioguanine in the liver. This metabolite inhibits the replication of DNA. It can also interfere with T-cell functions and B-cell antibody production. Therefore, AZA also affects several aspects of the immune system [23, 24].

We have listed a summary of treatment options and treatment outcomes of oral PV patients in studies published up to this point in Table 5 [8, 11, 12, 15, 16, 19, 25–36]. Combination therapy with various adjuvants has long been a subject of interest, and numerous studies have evaluated their efficacy. However, several adjuvants were often employed in one study [8, 11, 12, 26, 27, 32, 35]. As a consequence, differences in treatment outcomes cannot be attributed to the effect of an individual drug [17]. On the other hand, as shown in Table 5, the original studies were mostly confined to a small number of cases, which makes it difficult to evaluate the efficacy of a treatment option for this disease.

The European Academy of Dermatology and Venereology has recently published an important clinical practice guideline for PV management [37]. According to this guideline, first-line therapy includes: Rituximab: two infusions of 1 g two weeks apart, alone, or associated with oral prednisone 0.5 mg/kg/day with a rapid decrease to stop corticosteroids after 3 or 4 months. Systemic corticosteroids: prednisone 0.5–1.0 mg/kg/day with or without AZA (2.0 mg/kg/d), or mycophenolate mofetil 2 g/day or mycophenolate sodium 1440 mg/d. To note, rituximab is not universally available as a first-line treatment [37]. Our protocol is in line with this guideline in using low-dose corticosteroids with or without AZA which is more available universally.

It should be noted that screening for thiopurine methyltransferase (TPMT) activity is considered a baseline test to prevent unexpected myelotoxicity in patients receiving AZA. Nevertheless, the distributions of TPMT genotype and allele frequency in Iranian populations are different from the genetic profile found among Caucasian or Asian populations. TPMT^*∗*^2, TPMT^*∗*^3A, TPMT^*∗*^3B, and TPMT^*∗*^3C are inactivating alleles that explain 80% to 95% of individuals with lower TPMT activity. The most common subtypes such as TPMT^*∗*^3A and TPMT^*∗*^3C showed less frequency in our population according to different studies [38, 39]. Furthermore, results of previous Iranian studies revealed that there were no signs of both defective alleles in the population [38]. A study on Pemphigus Vulgaris patients taking AZA showed no TPMT^*∗*^2 or TPMT^*∗*^3C mutant alleles and none of the subjects developed hepatotoxicity and bone marrow suppression [39]. Therefore, we carefully monitored the patients' blood counts every month, and if signs of reduction in blood counts were observed, the TPMT test was ordered. Fortunately, in all 22 patients taking AZA, no significant changes in blood counts were observed.

### 4.1. The Use of AZA in Previous Studies

Many studies have indicated the favorable use of AZA in oral PV patients, although in most cases other immunosuppressive medications were used next to AZA [8, 11, 12, 15, 26–28, 31–33, 35] (Table 5).

Mignogna et al. treated 37 oral PV patients with a follow-up period of 2–13 years. For 28 patients, AZA was added to the standard treatment of corticosteroids. They reached complete healing of lesions in 34 of the patients in the mean time of 4.7 ± 2.57 months. Even though one patient died, the results were similar to our results [31]. In our study, all patients except one experienced complete remission in approximately 5 months.

Chaidemenos et al. compared two groups of patients with prednisolone 1.5 mg/kg/day and 40 mg on alternate days in combination with AZA. They reported that the group with combination therapy had the most treatment failure. Also, side effects were seen in both groups [19]. The difference observed in that study with this study can be due to the difference in the corticosteroid dosage used in the combination group. The same dosage was administered in both groups of our study.

Arduino et al. conducted a retrospective study on 98 oral PV patients. They used different adjuvants such as AZA, rituximab, and mycophenolate mofetil. They achieved complete remission in 80 patients in almost 4 months, but two patients died in their study [15]. The treatment protocol of this study is in contrast to our study which evaluated the effects of a single adjuvant next to corticosteroids with comparable results.

### 4.2. Treatment Outcome in Previous Studies

The current treatment protocol was effective for almost 98% of the patients who reached complete remission. Similar studies showed high favorable outcomes [11, 25, 26, 28, 29, 33, 35, 36]. In addition, we found much higher values for our treatment protocol compared to those reported by Scully et al. [27], Chaidemenos et al. [19], and Arduino et al. in 2019 [15] because contrary to their results, no death from PV or the treatment protocol had occurred.

A relapse occurred in 76.6% of our patients thereafter (generally one relapse during treatment). Scully et al. observed similar results with 76% relapse in their patients [27]. Other studies reported different results from 20% to 100% [30, 33, 35], while other studies noticed no relapse [12, 29, 34, 36]. The note to observe in studies with fewer relapses is the mean follow-up time which was less than a year in most studies, in comparison to our study which was around 4 years.

### 4.3. Treatment-Associated Side Effects

Although using systemic corticosteroids with adjuvants reduces the mortality rates, patients often experience considerable side effects of these treatments [24]. The most common side effects observed in our patients were hypertension, followed by gastrointestinal problems and insomnia. This is in line with other studies which reported the same side effects for corticosteroids and AZA administration [8, 11, 15, 19, 25–27, 29, 31]. In this study, the longer duration of treatment correlated with the presence of treatment-associated side effects, probably due to increased cumulative steroid dose throughout the treatment.

One of the most striking observations to emerge from the data comparison was the observation of significantly fewer side effects in patients receiving prednisolone in combination with AZA in comparison to the group receiving prednisolone alone. To the best of our knowledge, this comparison is not stated in other literature. The results showed that the time to achieve complete remission in patients treated with oral prednisolone plus AZA was approximately 30 days less than in patients treated only with oral prednisolone. Although this difference was not statistically significant, it may be clinically a possible explanation for significantly fewer treatment-associated side effects among patients treated with oral prednisolone in combination with AZA.

One of the particular strengths of our study is the long-term duration of the follow-up period. It is difficult to ascertain whether the treatment simply suppresses the manifestations of the disease or induces a long-lasting remission when long-term follow-up is not provided. Our results indicate both treatment regimens result in high rates of clinical response. But the comparison of both groups (prednisolone vs prednisolone plus AZA) is not statistically relevant. Randomization was not conducted in this study to administer the treatment regimens. Nevertheless, we hope our research will serve as a base for future long-term, randomized studies to compare other treatment protocols and other immunosuppressive drugs with AZA. There are numerous immunosuppressive medications. Choosing the most effective with the correct dosage can be a challenge for clinicians. Similar reports like this study can pave the way for the better management of chronic diseases like oral PV.

## 5. Conclusions

To conclude, according to our results, low-dose prednisolone in combination with AZA is an effective treatment regimen for oral PV patients. The use of adjuvant therapy with AZA can avoid the increase of corticosteroid dosage, thus reducing the treatment-associated side effects in severe cases. All patients should be observed closely in frequent follow-up sessions for treatment outcome, corticosteroid dose reduction, and treatment side effects. When high-dose corticosteroids are contraindicated or when rituximab is not available, this treatment protocol can guide clinicians in managing oral PV patients. To the best of our knowledge, our study had one of the largest groups of oral PV patients with long-term follow-ups who used one single protocol for all patients. Future cohort studies with a higher number of patients and longer follow-ups are suggested for the evaluation of treatment outcomes and side effects.

## Figures and Tables

**Table 1:** Protocol for oral prednisolone tapering in oral PV patients.

Step	No. of weeks	Oral prednisolone dose
1	4–6	1 mg/kg every day
2	4	40 mg every day
3	4	40 mg and 20 mg on alternate days
4	4	30 mg and 20 mg on alternate days
5	4	30 mg and 10 mg on alternate days
6	4	30 mg on alternate days
7	4	20 mg on alternate days
8	4	15 mg on alternate days
9	4	10 mg on alternate days
10 (remission)	During remission	5 mg on alternate days as maintenance dose

**Table 2:** Demographic, medical, and clinical characteristics of patients.

Group		Prednisolone	Prednisolone plus AZA	Total	*P* value^*∗*^ chi-square test
Gender	Male	7 (28.0%)	9 (40.9%)	16 (34.0%)	0.351
	Female	18 (72.0%)	13 (59.1%)	31 (66.0%)	

Age at baseline (years), mean value ± SD (range)		40.8 ± 14.529 (20 − 75)	43 ± 9.971 (27 − 60)		0.462

Job	Occupied	11 (44.0%)	9 (40.9%)	20 (42.6%)	0.831
	Unoccupied	14 (56.0%)	13 (59.1%)	27 (57.4%)	

Disease severity at baseline	1	14 (56.0%)	6 (27.27%)	20 (42.6%)	0.035^*∗*^
	2	9 (36.0%)	8 (36.36%)	17 (36.17%)	
	3	2 (8.0%)	8 (36.36%)	10 (21.27%)	

Number of patients with extraoral involvement (%)		31 (65.95)	14 (56)	17 (77.27)	
Systemic disease (%)		18 (38.29)	12 (48)	6 (27.27)	
Medications (%)		20 (42.55)	12 (48)	8 (36.36)	
Total		25 (53.2%)	22 (46.8%)	47 (100%)	

^*∗*^A *p*-value 0.05 was considered significant.

**Table 3:** Comparison of the clinical characteristics and treatment outcomes of the two treatment groups.

		Treatment groups		Total	*P* value^*∗*^
Number of follow-up sessions mean value ± SD (range)		20.76 ± 13.217 (2 − 48)	17.55 ± 9.951 (3 − 43)	19.201 ± 11.435	0.543
Number of patients who achieved a complete remission during treatment (%)		25 (100)	21 (95.45)	46 (97.87)	0.645
Number of patients relapsed after remission during treatment (%)	One relapse (%)	11 (44)	12 (54.5)	23 (48.93)	0.108
	Two relapses (%)	10 (40)	3 (13.6)	13 (27.65)	
Number of patients who underwent intralesional injection therapy during treatment (%)		8 (32)	11 (50)	19 (40.42)	0.210
Number of patients in complete remission at the last clinical evaluation (%)		13 (52)	13 (59.1)	26 (55.31)	0.626
Number of patients in partial remission at the last clinical evaluation (%)		12 (48)	8 (36.36)	20 (42.55)	0.257
Time to achieve a complete remission (days) mean value ± SD (range)		163.96 ± 292.984 (19 − 495)	134.24 ± 97.262 (12 − 404)	150.39 ± 224.075 (12 − 495)	0.343
The oral prednisolone dose at the time of complete remission (mg) mean value ± SD (range)		38 ± 18.428 (52 − 70)	36.19 ± 17.241 (5 − 70)	37.17 ± 17.722 (5 − 70)	0.642
The oral prednisolone dose at the last follow-up (mg) mean value ± SD (range)		13.70 ± 17.773 (1 − 65)	9.59 ± 10.065 (1 − 40)	11.78 ± 14.675 (5 − 65)	0.854
The oral prednisolone dose at the time of relapse(mg) mean value ± SD (range)		11.79 ± 15.169 (0 − 60)	6.88 ± 6.592 (0 − 20)	9.82 ± 12.567 (0 − 60)	0.538
Total (%)		25 (53.19)	22 (46.80)	47 (100)	—

^*∗*^A *p*-value 0.05 was considered significant.

**Table 4:** Adverse effects of treatment in oral PV patients.

Treatment complications	Group		Total	
	Prednisolone	Prednisolone plus AZA group		
With	20 (80.0%)	11 (50.0%)	31 (66.0%)	0.030^*∗*^
Without	5 (20.0%)	11 (50.0%)	16 (34.0%)	
Mild hypertension	7 (28%)	5 (22.72%)	14 (29.78%)	
Gastrointestinal problems	5 (20%)	6 (27.27%)	11 (23.40%)	
Insomnia	4 (16%)	3 (13.63%)	7 (14.89%)	
Liver dysfunction	2 (8%)	2 (9.09%)	4 (8.51%)	
Acne	4 (16%)	2 (9.09%)	6 (12.76%)	
Osteoporosis	1 (4%)	1 (4.54%)	2 (4.25%)	
Facial edema	4 (16%)	2 (9.09%)	6 (12.76%)	
Others	4 (16%)	3 (13.63%)	7 (14.89%)	
Total	25 (53.2%)	22 (46.8%)	47 (100.0%)	

^*∗*^A p-value <0.05 was considered significant.

**Table 5:** A summary of previous case series on the treatment of oral PV.

Study	Type and dose of corticosteroid	Type and dose of adjuvant	Outcome	Treatment side effects
Lozada et al. [25] FU: 19 months F/M: 3/3 *N* = 6	Systemic: (*N* = 6) *Pred* = 40–80 mg/day. Then the dosage gradually tapered due to the addition of *Lev*.	(*N* = 6) *Lev* = 100–200 mg/week	All of the patients responded (100%) within 2–8 weeks. CR = 3 (50%) PR = 3 (50%)	*Pred*: All of the patients had developed Pred adverse effects before adding Lev. *Lev*: chills, general malaise
Lamey et al. [26] FU: (5–20 yrs) F/M: 20/10 *N* = 30	Systemic: (*N* = 29) *Pred* = 20–120 mg/day. Then the dosage tapered to 10 mg daily on alternate days. Topical: (*N* = 1) *BM* for maintenance	(*N* = 5) *AZA* = 100 mg/day *CyclP* = 50 mg/day (*N* = 1) *GST* = 30 mg/week intramuscularly, subsequently extended to every 4 weeks	CR on therapy = 27 (90%) patients within 4–8 weeks of the start of therapy	*Pred*: Mild cushingoid face, epigastric discomfort due to peptic ulcer, diabetes mellitus, anxiety, fluid retention, insomnia, and hypertension
Robinson et al. [8] FU: mean = 4.5 years F/M: 9/3 *N* = 12	Systemic: (*N* = 11) *Pred* = at an initial dose of 50 mg/q.o.d–80 mg/day for 2–6 weeks, tapered according to disease response. Topical: (*N* = 11) *Fluocinonide, CB* or *Halobetasol* with Orabase (*N* = 4) *Dexamethasone* elixir 5 ml 3-4 times daily. (*N* = 1) *Betamethasone/Clotrimazole*	(*N* = 9) *AZA* = 25–150 mg/day (*N* = 6) *Lev* = 50–350 mg/week (*N* = 1) *MTX* = 2.5–15 mg/day (*N* = 1) *CyclP* = 15 mg/day (*N* = 1) *Dapsone*	CR = 9 (75%) PR = 3 (25%) Relapse during maintenance phase = 10 (83.33%) (mean = 0.20 relapse/year)	*Pred*: cushingoid features, weight gain, increased appetite, fluid retention, acute psychosis, headache, diabetes, depression, insomnia, fatigue, constipation, mood changes, acne, mild osteoporosis. *AZA*: GI upset, increased liver enzymes. *Lev*: malaise, itching. *MTX*: hair loss *Dapsone*: myelosuppression. *Topical steroids*: candidiasis, hairy leukoplakia
Scully et al. [27] FU: At least 3 months F/M: 33/22 *N* = 55 (follow-up data available for 32)	Systemic: (*N* = 28) *Pred* = 60 mg/day (range; 20–80 mg/day) Topical: (*N* = 32) Topical corticosteroids such as *BM*	(*N* = 17) *AZA* = 1–3 mg/kg/day (*N* = 3) *MTX* = 10–25 mg/week (*N* = 4) *Dapsone* = 50 mg/day (*N* = 1) *CyclP* = 50 mg/day	4 patients received topical corticosteroid alone. Systemic corticosteroid: CR = 5 (18%) Relapse = 21 (76%) Death = 2 (one as a consequence of immunosuppressive therapy).	*Pred*: lethargy, cushingoid faces, adrenal suppression, oral candidiasis, mild hypertension, diabetes mellitus, arthralgia, ankle swelling, easy bruising, cramps, osteoporosis, glaucoma, insomnia, CHF, acne, herpes zoster infection. *AZA*: abdominal pain, drug eruption, leukopenia, abnormal liver function, and vomiting. *MTX*: anemia, provoked nausea *Dapsone*: mild anemia
Mignogna et al. [28] FU: several years F/M: 11/5 *N* = 16	Systemic: (*N* = 14) *DZ* = 120 mg/day for 2–4 weeks (100 mg of *Pred* equivalent) then the dosage tapered to 6 mg every other day. (*N* = 3) *MT* = Pulse therapy at a dose of 1 grain daily for 3 days repeated after 21 days Topical: (*N* = 1) *CB* with Orabase (*N* = 1) Intralesional *TA*	(*N* = 7) *AZA* = 50–100 mg/day (*N* = 1) *CyclP* = 50 mg/day	Remission occurred in all cases (100%). Relapse during maintenance phase: One relapse = 4 (25%) Two relapses = 2 (12.5%) Three relapses = 1 (6.25%)	*DZ*: insomnia, hyperglycemia, increased appetite, mood changes, cataract, thrombocytopenia, osteoporosis, weight gain, hypercholesterolemia, biliary stasis, psychosis, fluid retention, hypokalemia, myopathy, cushingoid, headache, hypertrichosis. *MT*: flushing, insomnia, mood changes, hypertransaminasemia. *CB*: candidiasis. *AZA*: erythropenia
Femiano et al. [29] FU: NS F/M: 12/8 *N* = 20	Systemic: Group A: (*N* = 10) *Pred* = 125 mg/day, tapered to 5 mg once a week for 1 month. Group B: (*N* = 10) *Pred* = 50 mg/day, tapered to 5 mg/day once a week plus: *IV BM* = 5 cycles at intervals of 3 weeks	None	Clinical resolution: Group A = 30 days Group B = 25 days Subjective resolution of symptoms: Group A = 15 days Group B = 12 days	Gastritis, hyperglycemia, hypertension, increased body weight, mood change, altered calcium/phosphate metabolism
Camacho Alonso et al. [36]FU: NS F/M: 10/4 *N* = 14	Systemic: (*N* = 12) *Pred* = 60 mg/day, 1 month Topical: (*N* = 14) *TA* oral susp tid (*N* = 1) *Paramet* intralesional every 15 days, for 45 days	None	All patients responded to treatment, with lesion improvement at the end of therapy.	NS
Yazganoglu et al. [30] FU: 2–4 years F/M: 2/3 *N* = 5 (follow-up data available for 4)	Systemic: (*N* = 4) *MT* = 1-2 mg/kg/day	(*N* = 1) *MMF* = 2 g/day (*N* = 1) *Dapsone* = 50 mg/day	All of the patients showed at least one relapse. They were controlled with *MT* (3 patients) and *MT* and *MMF* in one.	*MT*: cushingoid appearance, and acneiform eruption. *MMF*: None
Azizi and Lawaf [16] FU: 3 months F/M: 15/5 *N* = 20	Systemic: (*N* = 20) *Pred* = 40 mg/day for 4 weeks Patients with moderate to severe disease started adjuvant therapy.	(*N* = 15) *Dapsone* = low initial dose gradually increased every week for 4 weeks. If new lesions developed, increased by 25 mg/day weekly until lesion free.	CR = 8 (40%) PR = 7 (35%) No noticeable change = 5(25%) (no response to *Dapsone*.)	NS
Mignogna et al. [31] FU: 2–13 years) F/M: 24/13 *N* = 37	Systemic: (*N* = 37) *Pred* = 75–100 mg/day (DZ equivalent) gradually tapered. Topical: (*N* = 28) 2 g *MT* in 500 ml saline mouthwash 4 times a day (*N* = 11) *CB* with Orabase Intralesional (*N* = 18) *TA* = 10 mg/0.25 ml	(*N* = 28) *AZA* = 50–150 mg/day (*N* = 3) *CyclP* = 50–100 mg/day	CR off therapy = 13 (35.1%) CR on therapy = 21 (56.8%) PR = 2(5.4%) Died = 1(2.7%) Relapse during the follow-up period: One relapse = 7 (18.92%) Two relapses = 2 (5.41%) Mean time to achieve a CR: 4.7 ± 2.57 months	*Pred*: insomnia, cataract, osteoporosis, hyperglycemia, mood alterations, weight gain, folliculitis, cushingoid aspect, hirsutism, increase in appetite, myopathy, headache, hypertrichosis, psychosis, tremors, thrombocythemia, biliary stasis, flushing, osteopenia, hypertransaminasemia. *AZA*: GI upset, erythropenia, hypertransaminasemia, cystitis
Mignogna et al. [11] FU: mean = 5.3 years F/M: 22/13 *N* = 35	Systemic: (*N* = 35) *Pred* = 75–100 mg/day (*DZ* equivalent) tapered to 50 mg twice a week. Topical PITA: (*N* = 16) Peri or intralesional *TA* = 40 mg/ml diluted 2 : 1 with saline per four lesions at weekly interval for 2–8 weeks No PITA: (*N* = 27) 2 g *MT* in 500 ml saline as a mouthwash (*N* = 10) *CB* with Orabase	PITA: (*N* = 13) *AZA* = 50–150 mg/day (*N* = 1)*CyclP* = 50–100 mg/day No PITA: (*N* = 14) *AZA* = 50–150 mg/day (*N* = 1) *CyclP* = 50–100 mg/day	CR at last control = 35 (100%) CR off therapy at last control = 13 (37.1%) CR on therapy at last control = 21 (60%) Died = 1 (2.9%)	PITA: candidiasis, yellowish gingival pellets, and gingival neo-vascularization. No PITA: candidiasis
Chaidemenos et al. [19] FU: at least 24 months F/M: 20/16 *N* = 36	Monotherapy: (*N* = 17) *Pred* = 1.5 mg/kg/day Lever's mini treatment: (*N* = 19) *Pred* = 40 mg on alternate days	Monotherapy: None Lever's mini treatment: (*N* = 19) *AZA* = 100 mg/day	CR on therapy [mean time to achieve]: Monotherapy = 9 (60%) [119.67 days] Lever's = 7 (46.7%) [234.47 days] CR off therapy: Monotherapy = 1 (6.7%) Lever's = 2 (13.3%) PR on therapy: Monotherapy = 3 (20%) Lever's = 4 (26.7%) PR off therapy: Monotherapy = 2 (13.3%) Lever's = 2 (13.3%) Treatment failure: Monotherapy = 1 (6%) Lever's = 4 (21%)	Monotherapy: weight gain, deregulation of glucose serum levels, redistribution of body fat, hypertension, eye disease, psychological effects, muscle weakness, GI disturbance, arrhythmias, liver enzymes` elevation, internal infection, hematologic toxicity, death. Lever's mini: deregulation of glucose serum levels, liver enzymes` elevation, weight gain, redistribution of body fat, hematologic toxicity, muscle weakness, GI disturbance, hypertension, psychological effects, hair loss, eye disease, internal infection
Ojaimi et al. [32] FU: NS F/M: 12/9 *N* = 21	Systemic: (*N* = 21) *Pred* = approximately 1 mg/kg/day Then tapered according to clinical activity	*AZA* = 1 mg/kg/d, escalating to 2 mg/kg/d after 2 weeks *MMF* = 1 g/day in two doses, after a fortnight. 2 g/day; in suboptimal response 3 g/day. *RTX* = four doses of 375 mg/m^2^, given weekly or two doses of 1 g given a fortnight apart. Cohort 1: (*N* = 3) *MMF* (*N* = 1) *RTX* Cohort 2: (*N* = 13) *AZA*, (*N* = 12) *MMF* (*N* = 11) *RTX* Cohort 3: (*N* = 5) *MMF* (*N* = 2) *RTX*	Cohort 1: Remission on *MMF* = 2 Remission after a single dose of *RTX* = 1 Cohort 2: Long-term disease control by *AZA* = 1 Remaining were changed to *MMF*. Cohorts 2 and 3: Disease control with *MMF* = 4. *RTX* = controlled off steroids = 11 CR = 1 Initially controlled but relapsed = 7.	*AZA*: hypersensitivity reaction (fever and rash) and abnormal liver function tests. *MMF*: gastrointestinal symptoms.
Vinay et al. [33] FU: 5-6 months F/M: 2/1 *N* = 3	Systemic: (*N* = 3) Premedication with IV *hydrocortisone* = 100 mg Topical: (*N* = 2) *Pred* = 0.5 mg/kg/day as a concomitant therapy of intralesional *RTX* injections. Tapered and stopped by month 5.	(*N* = 3) Intralesional *RTX* injection = 10 mg/ml, used in 5 mg/cm^2^ injections on days 1 and 15 (*N* = 1) *AZA* = 1.5 mg/kg/day as a concomitant therapy	All the patients achieved CR (100%). Relapse during the follow-up period = 1 (33.33%)	*RTX*: Pain during intralesional injections, lasting for 2-3 days
Sultan et al. [34] FU: range: 2–260 weeks F/M: 20/11 *N* = 31 (follow-up data only available for 20)	Systemic: (*N* = 20) *Pred* = 0.5–1 mg/kg Topical: (*N* = 17) *Dexa* rinses (*N* = 1) *CB* rinses (*N* = 1) *Fluocinonide* gel (*N* = 3) *CB* gel (*N* = 1) Intralesional *TA*	(*N* = 7) *MMF* = 500–1000 mg twice a day (*N* = 1) *RTX* infusion = 375 mg/m^2^ (*N* = 1) *Dapsone* = 50 mg four times a day *IVIg*	Control on therapy at first follow-up = 16 of 20 patients (80%) Control at the second follow-up visit = 4 (20%)	*Corticosteroid*: oral candidiasis. *IVIg*: anemia
EL-Komy et al. [12] FU: 90 days F/M: 7/4 *N* = 11	Systemic: (*N* = 11) IV *MT* for three successive days every 2–4 weeks Topical: (*N* = 11) One buccal mucosa was injected with 10 mg/ml *TA*, and the other buccal mucosa was injected with 1 ml of autologous *PRP* every 14 days.	(*N* = 11) *CyclP* intravenous pulse therapy = 1000 mg once a month (*N* = 9) *AZA* = 100–200 mg/day (*N* = 2) *MMF* = 2 g/day	The mean clinical improvement: *TA* injected area: 61.25% *PRP* injected area: 62.25% Both intralesional *PRP* and *TA* helped improve the oral erosions and pain in 7 patients (78%).	None
Arduino et al. [15] FU: mean ± SD = 85.12 ± 59.35 months F/M: 61/37 *N* = 98	Systemic: (*N* = 95) *Pred* = 1–1.5 mg/kg/day usually for 2–4 months. The dosage gradually tapered until a maintenance dose of 2.5 mg every 2, 3, or 4 days and then stopped. Topical: (*N* = 66) *CB* = 0.05% ointment	(*N* = 48) *AZA* = 2 mg/kg (*N* = 16) Intravenous *RTX* = 1000 mg on days 0 and 14 (*N* = 13) *MMF* = 1.5–2 g/day (*N* = 3) *IVIg* = 4 monthly cycles at a dose of 2 g/kg /cycle (*N* = 1) *Dapsone* = 50 mg/day	Three patients controlled their disease only by using topical corticosteroids Systemic treatment: CR off therapy = 39 (41.05%) CR on therapy = 41 (43.15%) PR on therapy = 15 (15.8%) Died = 2 (2.10%) Mean time to achieve a CR: 3.9 ± 2.72 months	*Pred*: myocardial infarction, femoral fracture, weight gain, and cushingoid features. *Adjuvants*: osteoporosis, cardiovascular problems, hyperglycemia, mood disorders, gastric disease, cataract, pneumonia, walking problems, hair loss, folliculitis, tremor, hypokalemia, erythroderma.
Fortuna et al. [35] FU: mean ± SD = 57.2 ± 37.7 weeks F/M: 7/3 *N* = 10	Short-term duration of the *corticosteroid* therapy. Additional details were not mentioned.	(*N* = 10) *RTX* infusion = 375 mg/m^2^ once weekly for 4 weeks an additional 1–4 monthly based on the patient`s clinical conditions. (*N* = 5) *AZA* (*N* = 3) *SFZ*	CR off therapy = 10 (100%) Relapse = 2 (20%) Mean time to achieve a CR: 19.8 ± 10.3 weeks	*Corticosteroid*: cushing's syndrome, osteoporosis, folliculitis, paroxysmal atrial fibrillation, GI issue, fatigue, insomnia. *RTX*: Infusion–related side effects

FU, follow-up period; F, female; M, male; Pred, prednisone; Lev, levamisole; CR, complete remission; PR, partial remission; NS, not stated; BM, betamethasone; AZA, azathioprine; CyclP, cyclophosphamide; CB, clobetasol; GST, Gold sodium thiomalate; MTX, methotrexate; DZ, deflazacort; MT, methylprednisolone; TA, triamcinolone acetonide; MMF, mycophenolate mofetil; PITA, perilesional/intralesional triamcinolone acetonide; RTX, rituximab; IVIg, intravenous immunoglobulin; PRP, Platelet-rich plasma; SFZ, sulfasalazine; Paramet, Paramethasone; tid, three times daily.

## Data Availability

The data supporting the findings of this study are available from the corresponding author upon reasonable request. However, restrictions are applied to the public availability of these data because of the patient's confidentiality.

## Abbreviations

PV: Pemphigus Vulgaris
AZA: Azathioprine.
